# The internal plumbing of algal chloroplasts

**DOI:** 10.7554/eLife.05983

**Published:** 2015-01-13

**Authors:** Moritz Meyer, Howard Griffiths

**Affiliations:** Department of Plant Sciences, Cambridge University, Cambridge, United Kingdom; Department of Plant Sciences, Cambridge University, Cambridge, United Kingdomhg230@cam.ac.uk

**Keywords:** Chlamydomonas, focused ion beam, cryo-electron tomography, chloroplast, thylakoid, Rubisco, other

## Abstract

High-resolution images of chloroplast structure in the alga Chlamydomonas offer new insights into photosynthesis.

**Related research article** BD Engel, M Schaffer, LK Cuellar, E Villa, JM Plitzko, W Baumeister. 2015. Native architecture of the *Chlamydomonas* chloroplast revealed by in situ cryo-electron tomography. *eLife* **4**:e04889. doi: 10.7554/eLife.04889

**Image** 3D structure of part of a chloroplast from the alga Chlamydomonas

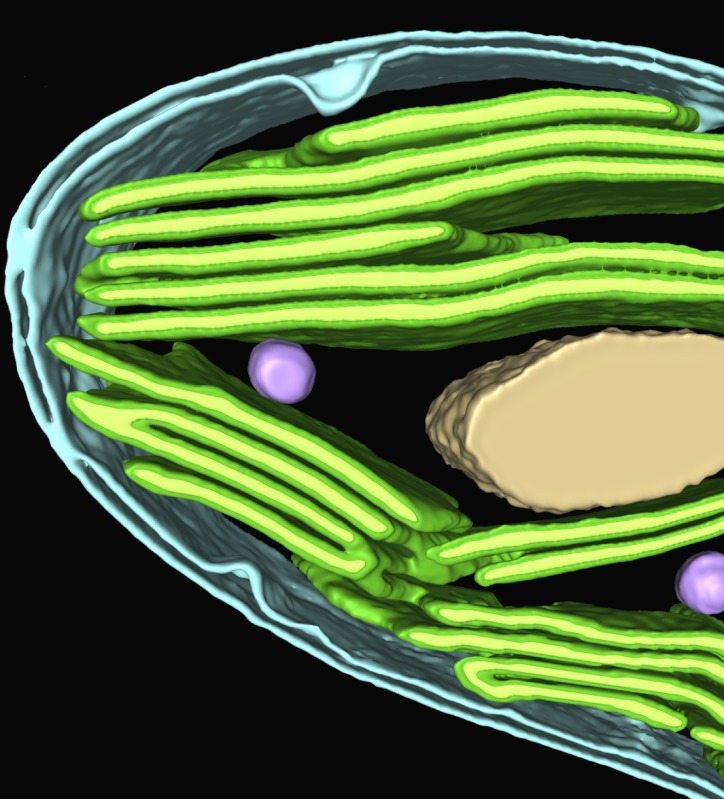

A soil-dwelling single-celled green alga called Chlamydomonas might not seem the most promising species to study when exploring the details of photosynthesis. There are more prominent groups of phytoplankton that can photosynthesize, but Chlamydomonas has become a ‘model’ eukaryotic microbe because it is simple to transform genetically and the genome has recently been sequenced.

In algal and plant cells, photosynthesis occurs within chloroplasts. Inside the double membrane that surrounds the chloroplast—known as the chloroplast envelope—light is harvested and converted into biochemical energy by pigment–protein complexes and associated proteins found in membrane structures called thylakoids. Loops of thylakoids merge to form an internal, acidified compartment known as the thylakoid lumen. The thylakoids are bathed in a fluid called the stroma, which contains enzymes that are involved in the fixation of carbon dioxide. Each Chlamydomonas cell has a single, cup-shaped chloroplast.

An enzyme called Rubisco catalyzes the crucial first step in carbon fixation and is found in all photosynthetic organisms. The rate of photosynthesis is limited by the amount of CO_2_ available to Rubisco: this is a particular problem in water because CO_2_ diffuses more slowly in water than in air and it is mostly found in the form of bicarbonate (HCO_3_^−^). To combat this problem, most microalgae—including Chlamydomonas—have evolved processes called carbon concentrating mechanisms that increase the amount of CO_2_ around Rubisco (Raven et al., 2014). These mechanisms enhance the efficiency of photosynthesis in three ways: the active transport of bicarbonate into the chloroplast; the conversion of bicarbonate back to CO_2_ by an enzyme called carbonic anhydrase; and the packaging of Rubisco into a small area to minimize the leakage of CO_2_.

How does a carbon concentrating mechanism manage to accumulate CO_2_ concentrations some 50–1000 times higher than are found in the environment? In the past this was a difficult question to answer because details of chloroplast structure were missing. Now, in eLife, Benjamin Engel, Miroslava Schaffer, Wolfgang Baumeister and co-workers at the Max Planck Institute of Biochemistry have combined cryo-focussed ion beam milling and cryo-electron tomography to provide a high-resolution 3D reconstruction of the internal architecture of the Chlamydomonas chloroplast in a near-native state (Engel et al., 2015).

The observations show in exquisite detail how, at the base of the chloroplast, the tips of thylakoid membranes fuse with the inner membrane of the chloroplast envelope. This may allow proteins from outside the chloroplast to enter the thylakoids more easily. Also, openings through the stacks of thylakoids allow the stroma to be continuous throughout the chloroplast. Other insights include details of a structure called the eyespot, which enables the alga to move in response to light.

We already knew that most of the Rubisco was confined to a small area within the stroma—called the pyrenoid—in Chlamydomonas. The pyrenoid is usually surrounded by a dense sheath of non-overlapping starch plates. Extensions of thylakoid membranes, called tubules, pass through the pyrenoid (Figure 1A). This is quite different to the structures in cyanobacteria where Rubisco is found: these structures, which are known as carboxysomes, are separated from the membranes that harvest light, and they have a protein coat and internal scaffold to support the Rubisco and the carbonic anhydrase.

**Figure 1.**
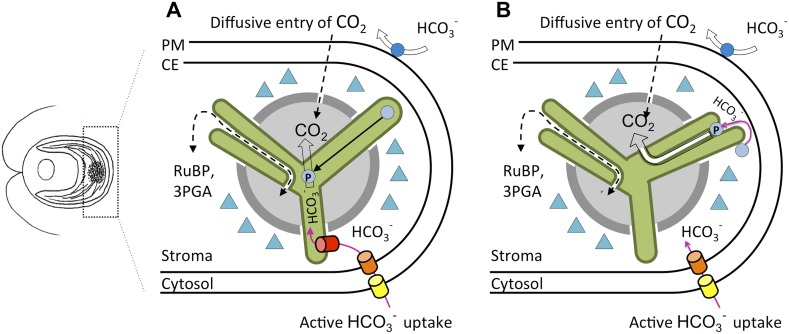
An updated carbon concentrating mechanism for Chlamydomonas. (**A**) Existing models of CO_2_ uptake in Chlamydomonas (Wang et al., 2011) allow for the diffusive entry of CO_2_, a process facilitated by a carbonic anhydrase, CAH1 (dark blue circle), in the periplasm. There is also a carbon concentrating mechanism that involves the transport of bicarbonate (HCO_3_^−^) across the plasma membrane (PM), possibly via the inorganic carbon pumps LCI1 and HLA3 (yellow barrel), and across the chloroplast envelope (CE), possibly via the inorganic carbon pump LCIA (orange barrel). The conversion of bicarbonate to CO_2_ occurs via CAH3 (light blue circles), a carbonic anhydrase thought to be located inside the lumen of thylakoid tubules (green). When CAH3 is phosphorylated it could relocate within the pyrenoid (light grey). The pump that transfers bicarbonate from the stroma to the lumen (red barrel) has not yet been found. CO_2_ is prevented from leaking out of the pyrenoid by the starch sheath (dark grey) and LCIB/C protein complexes (triangles). (**B**) Observations made by Engel et al. demonstrate that minitubules (white) provide a direct route between the stroma and the pyrenoid. This resolves how RuBP, which is the substrate for Rubisco (see text), is channelled to the enzyme, and how 3PGA, which is a product of the reaction between RuBP and CO_2_, is channelled away. It also raises the intriguing possibility that CAH3 could be external to the thylakoid lumen. When phosphorylated, CAH3 could relocate close to or within the minitubules, also allowing CO_2_ to be delivered via the minitubules to Rubisco without the need for an additional bicarbonate transporter.

The three-dimensional reconstructions of the pyrenoid show how the tubules extend from thylakoids at the interface of two starch plates, and how they fuse and then merge at the centre of the pyrenoid. Some of these features were visible in early electron micrograph images (Sager and Palade, 1957), but Engel et al. reveal how additional features called minitubules are organised within the tubules that cross the pyrenoid (Figure 1B). The minitubules contain stroma and the latest work clearly shows that they could provide routes for molecules to move from the stroma directly into the centre of the pyrenoid. This would allow substrates and products to move between Rubisco and the other enzymes involved in photosynthesis (Süss et al., 1995), and could reduce the need to transport bicarbonate across the thylakoid membranes, as is required in existing models (Wang et al., 2011).

Engel et al. also reveal how Rubisco is packaged in the pyrenoid. If their proposed model of close-packing in hexagonal shapes is exact, the spacing of Rubisco complexes leaves room for molecules of between 5 kDa and ~50 kDa, depending on the packaging scenario. The latter is sufficient space for a Rubisco linker protein like the one needed inside the carboxysomes of bacteria, but it is not clear whether such proteins also exist in algae (Long et al., 2007).

We can now superimpose these fine details onto existing models of how the carbon concentrating mechanism in Chlamydomonas is organized (Figure 1A). When the mechanism is switched on, bicarbonate could be converted by a stromal carbonic anhydrase within or adjacent to the minitubules (Figure 1B) to allow CO_2_ to be released at the heart of the pyrenoid.

This new model poses a number of questions. How does Rubisco move to the pyrenoid when the carbon concentrating mechanism is switched on? What is the trigger that modifies the thylakoid membranes to make the tubules as well as multiple minitubules? Why do the starch plates form and surround the pyrenoid? Efforts are being made to engineer elements of the carbon concentrating mechanism from cyanobacteria into higher plants (Lin et al., 2014; McGrath and Long, 2014), but what modifications would be needed to incorporate a mechanism from algae instead?

Undoubtedly, the many genetic techniques that can be used to study Chlamydomonas (Zhang et al., 2014) will soon resolve these questions in the case of this particular alga. However, in the best traditions of product placement, other forms of pyrenoid are available in different species, including phytoplankton and one lineage of primitive land plants. Understanding the structure of chloroplasts and the regulation of carbon concentrating mechanisms in these organisms will continue to provide additional challenges for the future.
